# Enhancement of the Antioxidant Activity of *Hedysari Radix* Particle Dispersion via ZIF-8/PEG Surface Co-Adsorption

**DOI:** 10.3390/molecules30234632

**Published:** 2025-12-02

**Authors:** Xionggao Han, Chaoyue Wang, Jianmei Wang, Qiqi Pan, Jinghui Feng, Guanglei Zuo

**Affiliations:** 1 Pharmaceutical Informatics Institute, College of Pharmaceutical Sciences, Zhejiang University, Hangzhou 310058, China; 2 Jinhua Institute of Zhejiang University, Jinhua 321016, China; 3 College of Pharmaceutical Engineering, Jinhua University of Vocational Technology, Jinhua 321016, China

**Keywords:** *Hedysari Radix* particles, co-adsorption modification, antioxidant potency, ZIF-8 co-incubation, medicinal plant resources

## Abstract

Herein, a co-adsorption modification based on ZIF-8 was introduced with the contribution of polyethylene glycol package to enhance the antioxidant potency of the Hedysari Radix disperse particles. In the solution system containing ethanol, the rough surface of the original Hedysari Radix disperse particles was improved by the typical co-adsorption modification with ZIF-8 and further smoothed by the package. The co-adsorption modifications, including ZIF-8 only, polyethylene glycol only, and ZIF-8 with polyethylene glycol, were all studied in the solution system. In particular, the modification that combined both the polyethylene glycol package and a suitable amount of ZIF-8 achieved the most significant enhancement of the catalase activity as well as the total antioxidant capacity value. The obtained hit platform alleviated the oxidative stress upon *Caenorhabditis elegans* and extended the survival time. This work suggested that meaningful co-adsorption modification could improve the potency of medicinal plant resources.

## 1. Introduction

Medicinal plant resources, which are usually used for preventing and treating diseases, are important medical resources worldwide [1,2]. The quality and potency bottleneck has brought the dilemma of high cost and low efficiency due to the sophisticated factors of natural growth, collection, planting, and processing [3,4]. Therefore, manufacturing supervision and product modification are significant for the successful utilization of the medicinal plant resources [5]. The former is commonly fixed by setting the corresponding standards, while the latter is more attractive due to the possible promotion of the given raw materials. Herein, we focused on the co-adsorption modification strategy, which commonly loaded the regular materials onto the traditional Chinese medicine particles, for improving the antioxidant potency. In particular, *Hedysari Radix* was selected as a representative.

*Hedysari Radix* is the dried root of *Hedysarum polybotrys* Hand. -Mazz and named for its ruddy skin [6]. It is moderately nutritious and sweet flavored. The main functions of *Hedysari Radix* include tonifying the life energy (also called “Qi” in the basic theory of traditional Chinese medicine), stabilizing surfaces, stopping perspiration, conducting detoxification, and contributing to diuresis and anti-inflammation [7,8,9]. Previous reports indicated that *Hedysari Radix* had a higher antioxidant potency among similar sources [10,11]. In clinical trials, *Hedysari Radix* has been used in the treatment of spontaneous sweating, blood obstruction, swelling, poor food intake, chronic diarrhea, hematochezia, bleeding, edema, and pain [12,13,14]. It also plays important roles in immune regulation, anti-inflammation, and carcinoma inhibition [15,16,17]. The main components of *Hedysari Radix,* such as polysaccharides, terpenoids and flavonoids (or isoflavones), all indicated considerable antioxidant effects, such as water decoction and extraction [18,19,20,21]. Among them, the typical phytochemicals included naringin, naringenin, daidzein, calycosin, betulinic acid, and lupeol. However, theoretical research of the traditional Chinese medicine requires an acceptable minimum size of 200 μm [22,23,24]. Since *Hedysari Radix* is investigated as a complete medicinal herb, the disperse particles of a suitable size (~200 μm) are more in line with the mentioned theoretical research [22].

To match the appropriate scale of the *Hedysari Radix* disperse particles, the co-adsorption modification should be designed with concern for size, dispersibility, target delivery, and biocompatibility. In recent decades, metal–organic frameworks (MOFs) have been introduced into the field of medicinal modification due to their specific steric properties [25,26]. They are a porous polymer with a regular topology [27]. Among the reported MOFs, zeolitic imidazolate frameworks (ZIFs) are of high interest due to their practical biocompatibility and high drug loading efficiency [28,29,30]. With both organic and inorganic properties, ZIFs are commonly pH responsive and display a nanopore size, which is beneficial for the delivery of both hydrophilic and hydrophobic drugs. For loading medicinal herbal disperse particles, the combination strategy with MOFs is more like a co-adsorption modification rather than the pore loading one [31]. Actually, the size of the traditional Chinese medicine or herbal disperse particles is commonly larger than MOFs; thus, the usual strategy is loading MOFs onto the herbal disperse particles to improve surface attributes such as solubility and affinity towards the targets, which is different from encapsulation or post-synthetic loading [32,33]. Several MOFs (such as ZIF-8 and Cu-based ones) showed antioxidant potency themselves [34,35,36], while loading onto biomacromolecules (such as bovine hemoglobin) enhanced the antioxidant properties, with the probable mechanism of improving the irregular surfaces [37]. Polyethylene glycol (PEG) package was further used to enhance the stability of the acquired particles, which also enhanced the antioxidant activity as referenced [38,39]. The enhancement of the antioxidant potency has been reported in the field of preparing traditional Chinese medicine or herbal disperse particles [40,41]. Generally speaking, developing rational co-adsorption modification, or similar strategies, may improve the antioxidant activity of *Hedysari Radix*.

**Figure 1:**
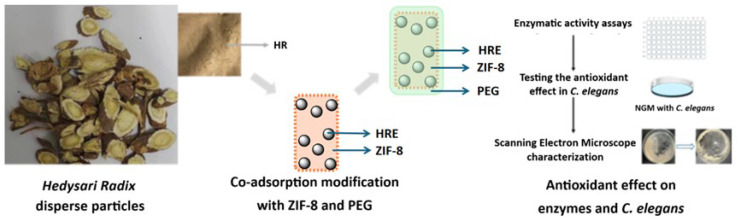
The co-adsorption modification with ZIF-8 and PEG upon the *Hedysari Radix* disperse particles to enhance the antioxidant potency.

In this work, a co-adsorption modification with ZIF-8, a typical MOF composed of Zn^2+^ and 2-methylimidazole, was carried out upon the *Hedysari Radix* disperse particles, together with the contribution of PEG package, to potentially enhance the antioxidant potency (Figure 1). The size of *Hedysari Radix* disperse particles was 200 μm, while the diameter of the ZIF-8 particles chosen from the commercial source was 5 μm. In consideration of the reported antioxidant activity and ability of both ZIF-8 and PEG package to improve the surface, ZIF-8 particles were loaded onto the surface of *Hedysari Radix* disperse particles via co-adsorption; this was expected to enhance the antioxidant potency according to the literature [40,41]. The prepared samples were characterized and tested to determine the structural modification. The antioxidant potency was also evaluated at the enzymatic level and in *Caenorhabditis elegans* (*C. elegans*). Most significantly, this work introduced co-adsorption modification, which was expected to improve the antioxidant potency with convenience and cooperativity. The strategy here was easy to operate and could be combined with other approaches. Generally, this work might raise the possibility of co-adsorption modification and broader strategies for improving the biological activity of medicinal plant resources.

## 2. Results and Discussion

### 2.1. Co-Adsorption Modification on the Hedysari Radix Disperse Particles

The chemical composition of *Hedysari Radix* was further characterized by a HPLC chromatogram (Figure S1), in which peaks 1–6 were assigned to ferulic acid, isoferulic acid, vanillic acid, ononin, formononetin, and formononetin-7-O-*β*-D-glucoside, respectively. Based on a comparison with the authentic standards, detailed composition information is summarized in Table S1. Initially, the solution system was studied to realize the co-adsorption modification. The *Hedysari Radix* powder (0.2 g) was added to the mixed water–ethanol solvent (10 mL). After the determination of the weight of the dissolved amount of *Hedysari Radix*, it was found that the percentage of ethanol in the mixed solvent at 15% was an efficient and suitable parameter (Figure S2). The corresponding dissolved amount was 48.35 ± 1.74% of the added powder. In this step, two samples were preliminarily acquired as *Hedysari Radix* powder in water (HRW) and *Hedysari Radix* powder in 15% ethanol (HRE). Then, in the following experiments, the ethanol percentage was set as 15%.

**Figure 2:**
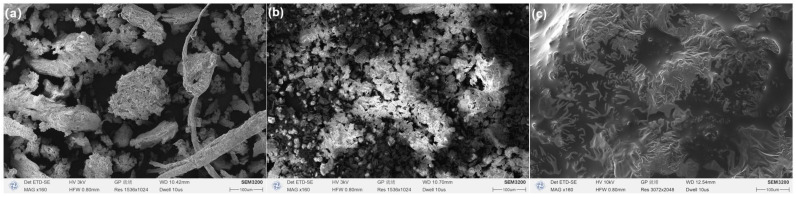
The images under scanning electron microscope of (**a**) HRE, (**b**) HRE@ZIF-8 3, and (**c**) HRE@ZIF-8@PEG 3. Scale bar: 100 μm.

Subsequently, in the prepared solvent system, different masses of ZIF-8 (5, 10, 20, and 40 mg) were added and mixed to acquire the ZIF-8-modified groups (HRE@ZIF-8 group: 1~4, from low dosage to high). One step further, the ZIF-8-modified groups were wrapped with PEG to form the PEG-modified groups (HRE@ZIF-8@PEG group: 1~4, from low dosage to high). One extra group was prepared as *Hedysari Radix* powder in 15% ethanol with PEG (HRE@PEG), which contained no ZIF-8. All samples were freeze-dried. The groups with equal material mass ratios (HRE, HRE@ZIF-8 3, and HRE@ZIF-8@PEG 3) were selected as representatives to check the basic characteristics under the scanning electron microscope (Figure 2). The HRE group exhibited an appropriate diameter of 200 μm and a rough surface with many gaps, which was suitable for disperse particles, following the theoretical research in the field of traditional Chinese medicine (Figure 2a). After the co-adsorption modification with ZIF-8, the ZIF-8 particles with an original diameter of 5 μm assembled into the gaps of HRE (Figure 2b). In this pattern, the ZIF-8 particles were loaded on the surface of the original HRE particles. The HRE@ZIF-8 3 group formed in this approach also showed an appropriate diameter of 200 μm, while the surface was not that rough. Further, the modification with PEG via the package onto the outer layer led to a smooth surface, which even blurred the boundaries of particles (Figure 2c). As we mentioned in the introduction, it was reported that the modification of the herbal medicine should keep the diameter over 200 μm. Herein, all the tested samples maintained a diameter in line with the theoretical guidance of traditional Chinese medicine and mainly provided a rational modification on the morphology of the surface, which might be more significant through antioxidant procedures.

### 2.2. Testing the Antioxidant Activity in the Solution System

Herein, to calibrate the background signals and ensure rational optical density (OD) values in the solution system, the tissue homogenate containing a microsomal enzyme was used to check the correlation between the OD values of the system and the homogenate concentration, as detailed in the experimental section. As a result, the OD values increased along with the increase in the homogenate concentration in the range of 0–10% (Figure S3). To keep the OD values as 0.2–0.7 (according to the Lambert–Beer law), the homogenate concentration was set as 10% in the following tests.

The mechanism, modifying the *Hedysari Radix* disperse particles with either MOFs or PEG, might improve surface attributes such as solubility and affinity towards the targets, thus enhancing the antioxidant potency. On the other hand, the selected ZIF-8 itself had some antioxidant activity [37,42]. Distinguished from previous reports by loading small molecules inside ZIF-8 [43], the co-adsorption modification in this work maintained the internal loading capacity for introducing more functions.

**Figure 3:**
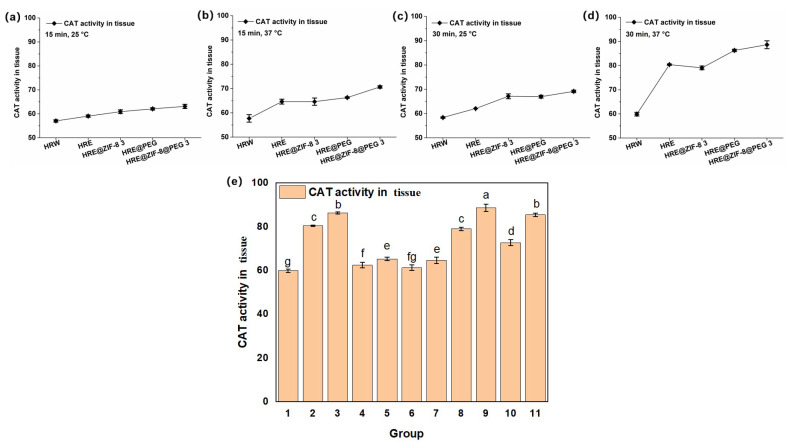
The CAT activity (U/mg) under different incubation temperature and time conditions: (**a**) 15 min, 25 °C; (**b**) 15 min, 37 °C; (**c**) 30 min, 25 °C; (**d**) 30 min, 37 °C; (**e**) 11 groups of samples, 30 min, 37 °C; 1–11 groups corresponding to: (1) HRW; (2) HRE; (3) HRE@PEG; (4) HRE@ZIF-8 1; (5) HRE@ZIF-8@PEG 1; (6) HRE@ZIF-8 2; (7) HRE@ZIF-8@PEG 2; (8) HRE@ZIF-8 3; (9) HRE@ZIF-8@PEG 3; (10) HRE@ZIF-8 4; (11) HRE@ZIF-8@PEG 4.

Subsequently, the effect of the samples on the activity of catalase (CAT), which catalyzes the decomposition of hydrogen peroxide and is present at high concentrations in the liver, was tested on the basis of the above solution system. In consideration of setting the orthogonal conditions, a different incubation time (15 and 30 min) and temperature (25 and 37 °C) were selected, while an OD value of 405 nm was set as the testing index. As an exploratory trail, five main groups of samples, including HRW, HRE, HRE@ZIF-8 3, HRE@PEG, and HRE@ZIF-8@PEG 3, were involved in the tests of the orthogonal conditions. After the reaction at 25 °C for 15 min, HRE obviously improved the CAT activity from HRW with the water solvent; meanwhile, the modification with ZIF-8 only (HRE@ZIF-8 3), PEG only (HRE@PEG), and ZIF-8 with PEG (HRE@ZIF-8@PEG 3) raised the CAT activity more notably (Figure 3a). When the incubation time remained 15 min and the temperature was raised to 37 °C, the effect of HRE was further enhanced (Figure 3b). In this condition, the individual modification with ZIF-8 (HRE@ZIF-8 3) did not show the obvious enhancement of CAT activity. Modification with PEG only (HRE@PEG) slightly enhanced the antioxidant effect of HRE. In comparison, the modification with both ZIF-8 and PEG (HRE@ZIF-8@PEG 3) indicated an obvious enhancement of the CAT activity. Under the conditions of 25 °C for 30 min, based on the fact that HRE showed a better effect than HRW, the modification with ZIF-8 (HRE@ZIF-8 3) or PEG (HRE@PEG) separately resulted in a remarkable increase in CAT activity, while the combination of ZIF-8 and PEG (HRE@ZIF-8@PEG 3) led to the further enhancement of the antioxidant effect (Figure 3c). Then, under the conditions of 37 °C for 30 min, the trend was similar to that under the conditions of 37 °C for 15 min (Figure 3d). Accordingly, several insights were revealed. The benefits offered by the solvent system were basically related to the temperature, while the enhancement enabled by the ZIF-8 modification should be maintained by extending the incubation time. Meanwhile, the PEG modification led to a positive affect by either increasing the temperature or extending the incubation time. The combination of ZIF-8 and PEG exhibited the most efficient improvement in the CAT activity in all the set conditions. Moreover, since the conditions of 37 °C for 30 min were the most able to raise the CAT activity, all eleven samples were tested in this system (Figure 3e). The labels “a–g” were added from significant to insignificant, as described in the experimental section. According to the results, in each pair of comparisons, the package of PEG enhanced the CAT activity compared to the original status. However, the ZIF-8 modification only enhanced the CAT activity at the suitable addition ratio (HRE@ZIF-8 3, and HRE@ZIF-8@PEG 3). Otherwise, the CAT activity was reduced. Thus, among all the tested samples, the modification combining both the PEG package and suitable amount of ZIF-8 resulted in the most significant enhancement of the CAT activity. Free from loading antioxidant agents or using MOFs with strong reducibility [44,45], the notable enhancement in the antioxidant potency here might be attractive due to the possibility of further improvement.

**Figure 4:**
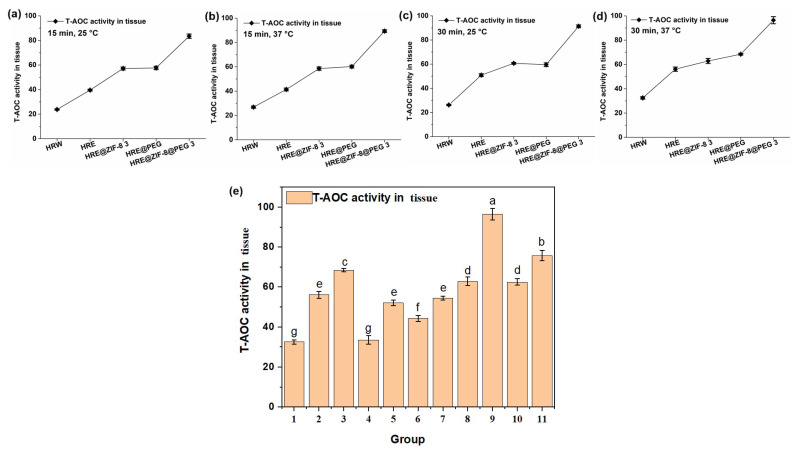
The T-AOC values (U/mg) under different temperature and time conditions: (**a**) 15 min, 25 °C; (**b**) 15 min, 37 °C; (**c**) 30 min, 25 °C; (**d**) 30 min, 37 °C; (**e**) 11 groups of samples, 30 min, 37 °C; 1–11 groups corresponding to: (1) HRW; (2) HRE; (3) HRE@PEG; (4) HRE@ZIF-8 1; (5) HRE@ZIF-8@PEG 1; (6) HRE@ZIF-8 2; (7) HRE@ZIF-8@PEG 2; (8) HRE@ZIF-8 3; (9) HRE@ZIF-8@PEG 3; (10) HRE@ZIF-8 4; (11) HRE@ZIF-8@PEG 4.

Afterwards, the total antioxidant capacity (T-AOC) value, which is an indicator of the quantity and activity of antioxidants in the body and reflects the body’s ability to combat free radicals and oxidative stress, was measured with the above solution system and orthogonal conditions (15 and 30 min; 25 and 37 °C). An OD value of 520 nm was set as the testing index. Similar to the measurement of CAT activity, the five main groups of samples, including HRW, HRE, HRE@ZIF-8 3, HRE@PEG, and HRE@ZIF-8@PEG 3, were involved in the tests of the orthogonal conditions. In all the tested conditions. such as 25 °C for 15 min (Figure 4a), 37 °C for 15 min (Figure 4b), 25 °C for 30 min (Figure 4c), and 37 °C for 30 min (Figure 4d), the observed tendencies were almost the same. On the basis of the fact that HRE showed a better effect than HRW, the modification with ZIF-8 (HRE@ZIF-8 3) or PEG (HRE@PEG) separately resulted in the significant enhancement of the T-AOC value to almost the same extent. In particular, the combination of ZIF-8 and PEG (HRE@ZIF-8@PEG 3) led to the dramatic enhancement of the T-AOC value compared with all the other groups. Then the conditions of 37 °C for 30 min were selected to check all eleven samples (Figure 4e). It was similarly suggested that in each pair of comparisons, the package of PEG increased the T-AOC value from the original status. The ZIF-8-modification, however, caused the T-AOC value to show a dose-dependent enhancement. With a low amount of ZIF-8, the T-AOC value was impacted, while with a high amount of ZIF-8, the T-AOC value was basically higher than that of HRE. Among all the tested samples, the modification combining both the PEG package and a suitable amount of ZIF-8 resulted in the most significant increase in the T-AOC value, which suggested a better antioxidant effect. Therefore, HRE@ZIF-8@PEG 3 was selected for the further investigation of the antioxidant effect upon *C. elegans*.

### 2.3. Testing the Antioxidant Activity upon C. elegans

**Figure 5:**
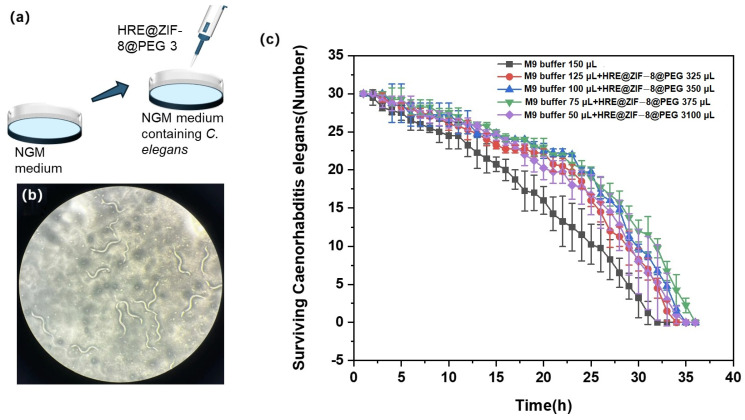
(**a**) The illustration of adding HRE@ZIF-8@PEG 3 in the L2 stage *C. elegans* to test the antioxidant effect; (**b**) The status of surviving *C. elegans* after the treatment of 1% hydrogen peroxide; (**c**) The survival time curves of the *C. elegans* under different incubation conditions with increasing volume of HRE@ZIF-8@PEG 3 solution (0 (black square), 25 (red dot), 50 (blue triangle), 75 (green inverted triangle), and 100 μL (purple diamond)).

In this section, L2 stage *C. elegans* was divided into six groups and treated with various conditions before being counted (Figure 5a). The control group was incubated with 150 μL of M9 buffer, while the other groups were incubated with an increasing volume of HRE@ZIF-8@PEG 3 solution (25, 50, 75, and 100 μL) and then supplied to reach a total volume of 150 μL with M9 buffer. In each group, 100 μL of 1% hydrogen peroxide was added every 2 h to induce oxidative stress, and the surviving *C. elegans* was checked under the microscope with the number counted (Figure 5b). The surviving number was recorded every hour until all the *C. elegans* died. The result is presented in Figure 5c. Basically, 32 h later, none had survived in the control group. The treatment with HRE@ZIF-8@PEG 3 solution extended the survival time of *C. elegans* under oxidative stress to 34–36 h. Compared with previous reports of similar models with marketed drugs such as Metformin and D-mannose [46,47], increasing the lifespan by 2–4 h in this work was notable. The antioxidant effect was mainly improved in a dose-dependent manner in a rational range (0–75 μL). When a higher dosage (100 μL) was given, the antioxidant effect was not the most potent but slightly weaker. In brief, the rational treatment of HRE@ZIF-8@PEG 3 was a workable strategy able to achieve a significant antioxidant effect upon *C. elegans* to alleviate oxidative stress and extend the survival time.

### 2.4. Discussion

The co-adsorption modification in this work retained the traditional Chinese medicine theory-accepted size of the raw *Hedysari Radix* disperse particles. The co-adsorption modification was beneficial for enhancing the antioxidant potency of the medicinal plant. The CAT activity and the T-AOC value were both improved. The oxidative stress of the *C. elegans* was alleviated and the survival time was extended. One of the most significant advantages of the co-adsorption modification here was the convenience. It was easy to operate with the simple components of ethanol, ZIF-8, and PEG. The previous antioxidant-enhancing strategies for medicinal plants were limited by the complex regulation of the levels of the components. Some of them performed the induction with natural or chemical agents to promote the growth and increase the content of the antioxidant compounds [48,49,50,51]; meanwhile, some others used irradiation to enhance the antioxidant potential [52,53]. The above approaches showed an impact on the relative contents of the components. Further strategies related to the product formation (such as infusions, essential oil) of the medicinal plants included gastro-duodenal digestion and *β*-cyclodextrin-based MOF [54,55]. They disrupted the overall integrity of the original medicinal plants, and the agents involved in optimization were relatively sophisticated. These approaches required a specific form of the medicinal plants, and were difficult to cooperate with other methods. It was notable that the co-adsorption modification in this work indicated practical cooperativity. The combination with other approaches and the introduction of function onto the MOF might lead to a more sophisticated performance. One obvious limit of this work was that the selection of ZIF-8 and PEG followed the necessity of convenience. Thus, further modification would have many stories to tell. For example, photodynamic therapy [56] and fluorescence labeling [57] might be introduced by adding functional molecules (such as indocyanine green) inside the cavity or onto the surface of the MOF. On the other hand, other MOFs such as UiO-66 or MIL-101 might alter ZIF-8 to offer catalysis and magnetism functions [58], while PEG could be replaced by other layers such as hyaluronic acid to affect the packaging performance [59]. On the basis of the findings here, more versatile functions might be enabled to achieve the accurate and effective utilization of medicinal plants.

## 3. Materials and Methods

### 3.1. General Materials and Instruments

The commercially available chemicals were purchased from Sangon Bioengineering Co., LTD. (Shanghai, China) and Sinopharm Group Chemical Reagent Co., LTD. (Shanghai, China). The raw *Hedysari Radix* was at the purity of 99%. ZIF-8 was at the purity of 98%, with a diameter of 5 μm. PEG was of reagent grade with an average molecular weight of ~900,000. They were directly used without further purification. The buffer solution was purchased from Sinopharm Group Chemical Reagent Co., LTD. (Shanghai, China). The ethanol used was at the purity of 95% at the reagent grade. The biological assay kits were purchased from Nanjing Jiancheng Biotechnology Co., LTD. (Nanjing, China). The kits for CAT activity and T-AOC (referring to the total antioxidant level composed of various antioxidants and antioxidant enzymes, such as vitamin C, vitamin E, and carotenoids) were used according to the common procedures in previous studies [60,61,62]. As a common choice, the tissue homogenate of the porcine liver was used as the hepatic microsomal enzyme-contained tissues. The *Hedysari Radix* was crushed into disperse particles with an FW177 crusher from Tianjin Stete Instrument Co., LTD. (Tianjin, China). The centrifugation in this work was conducted with an EBA200 high-speed centrifuge from Sigma Zentrifugen Co., LTD. (Munich, Germany). The morphology of the prepared samples was characterized by a Hitachi S-3400N Scanning Electron Microscope (SEM) system (Tokyo, Japan). The enzymatic activity assays were conducted on a Tecan Infinite 200 PRO Microplate Reader (Männedorf, Switzerland). The absorbance and optical diameters were set to suit the requirements of the enzymes.

### 3.2. Preparation of the Hedysari Radix Disperse Particles and the Modified Samples

The raw *Hedysari Radix* was put into the crusher and the homogenizer (at 10,000 rpm) to obtain a fine powder. The powder passed through the 120-mesh sieve. The aperture of the 120-mesh sieve was around 100 μm, while the rod-shaped *Hedysari Radix* disperse particles could pass through the sieve at the length of 200 μm, with the diameter of the passing surface being less than 100 μm. Then the powder was dried at 105 °C until constant weight. Then the powder passed through the 120-mesh sieve again for later use. The general preparation of the disperse particles followed the industrial quality control standards of traditional Chinese medicinal herbs [63,64]. Although they were not specific for the *Hedysari Radix* disperse particles, they were considered as the initial standards for the preparation in this work before the description of the detailed steps.

Following a conventional method in the field of traditional Chinese medicine, the preparation of the *Hedysari Radix* disperse particles was carried out in ethanol solution [65]. Thus, a rational water–ethanol proportion was obtained. The *Hedysari Radix* powder (0.2 g) was dissolved in 10 mL of mixed water–ethanol solvent and stirred at 30 rpm for 15 min. The mixture formed a stable colloidal solution. Then the mixture was filtered, washed by cold ethanol three times, and lyophilized. Slightly distinguished from the previous reference, the combination of the dissolved sediment and the supernatant was lyophilized, rather than merely the supernatant (mainly polysaccharide). Since almost the complete composition of *Hedysari Radix* was retained, the samples in this work were relevant to traditional Chinese medicine. The residue was dried at 100 °C until constant weight and then weighed after being cooled to room temperature. By adjusting the percentage of ethanol in the water–ethanol solvent (0–75%, and absolute ethanol), the dissolved amount of *Hedysari Radix* powder was tested by removing the residue and then weighing the vapored solution. The tests were in triplicate. Accordingly, the percentage of ethanol was set as 15% in the following experiments.

By setting the amount of *Hedysari Radix* powder as 20 mg, the dissolving system was set as 2 mL water–ethanol mixing solvent containing 15% ethanol. Then, different amounts of ZIF-8 (5, 10, 20, and 40 mg) were mixed in the above system to acquire the ZIF-8-modified groups. The ZIF-8 particles chosen were 5 μm, the most commonly used size, and were from a commercial source. The co-adsorption process was conducted in a period of 12 h at room temperature (25 °C). These groups were further treated with 200 μL PEG to form the PEG-modified groups. This step was also conducted in a period of 12 h at room temperature (25 °C). There were 11 samples: *Hedysari Radix* powder in water (HRW), *Hedysari Radix* powder in 15% ethanol (HRE), *Hedysari Radix* powder in 15% ethanol with PEG (HRE@PEG), ZIF-8-modified *Hedysari Radix* powder in 15% ethanol (HRE@ZIF-8 group: 1~4, from low dosage to high), and *Hedysari Radix* powder in 15% ethanol with the treatment of both ZIF-8 and PEG (HRE@ZIF-8@PEG group: 1~4, from low dosage to high). All samples were freeze-dried before further tests.

### 3.3. Enzymatic Activity Assays

Initially, 1 g of mashed porcine liver tissue containing microsomal enzyme was treated with 9 mL of saline. Then, the mixture was centrifuged at 2500 rpm for 10 min to remove the supernatant under ice bath conditions to obtain the 10% tissue homogenate. As referenced, the tissue homogenate was regarded as the enzyme in the following evaluation of the prepared samples and their impact on the CAT activity and T-AOC [60,61,62]. The samples were prepared in 15% ethanol. Right before the tests, the prepared samples were mixed with the tissue homogenate under different time (15 and 30 min) and temperature (25 and 37 °C) conditions.

The CAT activity was tested with a kit with the ammonium molybdate method [60,61,62]. The absorbance at 405 nm was tested with an optical diameter of 0.5 cm. In consideration of the sample volume, reaction time, and homogenate protein concentration, the CAT activity values were calculated. The tissue homogenate was added before the reaction in the testing groups to reduce the interference due to its own absorbance signal. In the control group, the tissue homogenate was added ad hoc to retain the background signal.

Meanwhile, the T-AOC was measured with a specific kit [60,61,62]. The absorbance at 520 nm was tested with an optical diameter of 1.0 cm. In consideration of the sample volume, total volume of the reaction system, reaction time, and homogenate protein concentration, the T-AOC values were calculated. The tissue homogenate was added before the reaction in the testing groups to reduce the interference due to its own absorbance signal. In the control group, the tissue homogenate was added ad hoc to retain the background signal.

### 3.4. Testing the Anti-Oxidant Effect in C. elegans

The nematode (here was *C. elegans*) was purchased from Nanjing NJUBio Co., Ltd. (Nanjing, China) and cultured in Xingzhi College according to the ethics principles for experimental animals of Zhejiang Normal University. The nematode growth medium (NGM) was rinsed with M9 buffer, and the *C. elegans* were collected in a 1.5 mL EP tube and naturally precipitated. The supernatant was discarded, and the precipitate was resuspended in M9 buffer. Then, 1 mL of lysate was added to the retained precipitate. The lysis time was set as 10 min to ensure full lysis. A further centrifugation of 1 min was conducted at 4500 rpm, and the supernatant was discarded. Then, the centrifugation was repeated another two times. Finally, the remaining lysate was washed away with M9 buffer and the eggs were precipitated. The eggs were incubated in NGM with *Escherichia coli* OP50 at 20 °C for 4 h, with the *C. elegans* possibly crawling on the moss.

The L2 stage *C. elegans* obtained by synchronization were randomly divided into six groups. The control group was treated with 150 μL of M9 buffer. The other groups were treated with various volumes of HRE@ZIF-8@PEG 3 solution (25, 50, 75, and 100 μL) in 15% ethanol. The total volume was set as 150 μL with the supplement of M9 buffer.

Then, the antioxidant model was built as referenced [65]. The treated *C. elegans* were cultured in an incubator at 20 °C for 48 h. From each group, thirty *C. elegans* were picked and placed in the corresponding marked dishes. Then, 1% hydrogen peroxide was used to induce oxidative stress. Each group was supplemented with 100 μL of 1% hydrogen peroxide every 2 h. When the *C. elegans* did not respond to the platinum wire touch, they were sacrificed. The surviving number of *C. elegans* in each group was recorded every hour until all the *C. elegans* died. The experiment was conducted in triplicate.

### 3.5. Statistical Analysis

The results were expressed as mean value ± SD (standard deviation). IBM SPSS Statistics 26 software was used for data processing. The differential analysis of multiple sets of data with the alphabet marking method was conducted here [66]. According to this method, initially, all the data groups were arranged in descending order based on the average values. The group with the largest average value was labeled with “a”. Then, the comparison was conducted with the other groups in sequence. The following groups were labeled with “a” if the difference was not significant. When a group with a significant difference appeared, it was labeled with “b”. Then, the comparison continued and the groups with an insignificant difference were labeled with “b”. When a group with a significant difference appeared, it was labeled with “c”. Then, the comparison continued until the group with the smallest average value was labeled with a letter. The labels “a–g” were added from significant to insignificant on the basis of the values in a homogeneous subset by setting the *p* value as 0.05. In brief, “a–f” meant that there was a significant difference (*p* < 0.05), while “g” meant that there was an insignificant difference. The alphabet marking method can visually display the differences between data groups with the rational arrangement.

## 4. Conclusions

To sum up, in this work, to improve the antioxidant potency of the *Hedysari Radix* disperse particles, a co-adsorption modification based on ZIF-8 was introduced with the contribution of PEG package. The co-adsorption modification was realized in the solution system containing 15% ethanol. The typical co-adsorption modification with ZIF-8 (HRE@ZIF-8 3) improved the rough surface of the original *Hedysari Radix* disperse particles (HRE), while the further package of PEG (HRE@ZIF-8@PEG 3) resulted in a smooth surface to blur the boundaries of particles. All the tested samples followed the theoretical guidance of herbal medicine to keep the diameter over 200 μm. After the comparison of the co-adsorption modification including ZIF-8 only, PEG only, and ZIF-8 with PEG, it was found that the modification combining both the PEG package and a suitable amount of ZIF-8 achieved the most significant enhancement of the CAT activity, as well as the T-AOC value. Furthermore, the typical hit platform HRE@ZIF-8@PEG 3 was tested to check the antioxidant effect upon *C. elegans*. As a result, by rationally applying HRE@ZIF-8@PEG 3, the oxidative stress was alleviated and the survival time of the *C. elegans* was extended. In future research, MOFs with functional molecules (such as photodynamic therapy [56], fluorescence labeling [57]) loaded inside might be modified onto herbal disperse particles to realize a more sophisticated performance. The information in this work was meaningful for developing practical further strategies that are compliant with the theory of traditional Chinese medicine.

## Data Availability

Data are available from the authors upon request.

## Supplementary Materials

The following supporting information can be downloaded at: https://www.mdpi.com/article/10.3390/molecules30234632/s1: Figure S1. HPLC chromatogram of *Hedysari Radix*; Table S1. Identified chemical components of *Hedysari Radix* extract by HPLC; Figure S2. Dissolved amounts of *Hedysari Radix* in different ethanol percentages (0–100%); Figure S3. Absolute OD plots of different homogenate concentrations.
