# Supplementary material for: Enhancement of the Antioxidant Activity of Hedysari Radix Particle Dispersion via ZIF-8/PEG Surface Co-Adsorption
Source: Molecules. 2025 Dec 2;30(23):4632. doi: 10.3390/molecules30234632 (PMC12692810; doi:10.3390/molecules30234632)
Supplement: Supplementary file 1 [file molecules-30-04632-s001.zip › molecules-3949181-supplementary.pdf]

Supporting Information

Enhancement of the Antioxidant Activity of Hedysari Radix Particle Dispersion via ZIF-8/PEG Surface Co-Adsorption

Xionggao Han [1,2,#], Chaoyue Wang [1,2,#], Jianmei Wang [3], QIqi Pan [3], Jinghui Feng [3,*] and Guanglei Zuo[1,2*]

[1] Pharmaceutical Informatics Institute, College of Pharmaceutical Sciences, Zhejiang University, Hangzhou 310058, China;

[2] Jinhua Institute of Zhejiang University, Jinhua 321016, China;

[3] College of Pharmaceutical Engineering, Jinhua University of Vocational Technology, Jinhua 321016, China.

[#] The authors contributed equally.

Corresponding Authors:

First and last name: Guanglei Zuo, Jinghui Feng

E-mail addresses: guangleizuo@zju.edu.cn (G.Z.)； jhfeng13@163.com (J.F.)

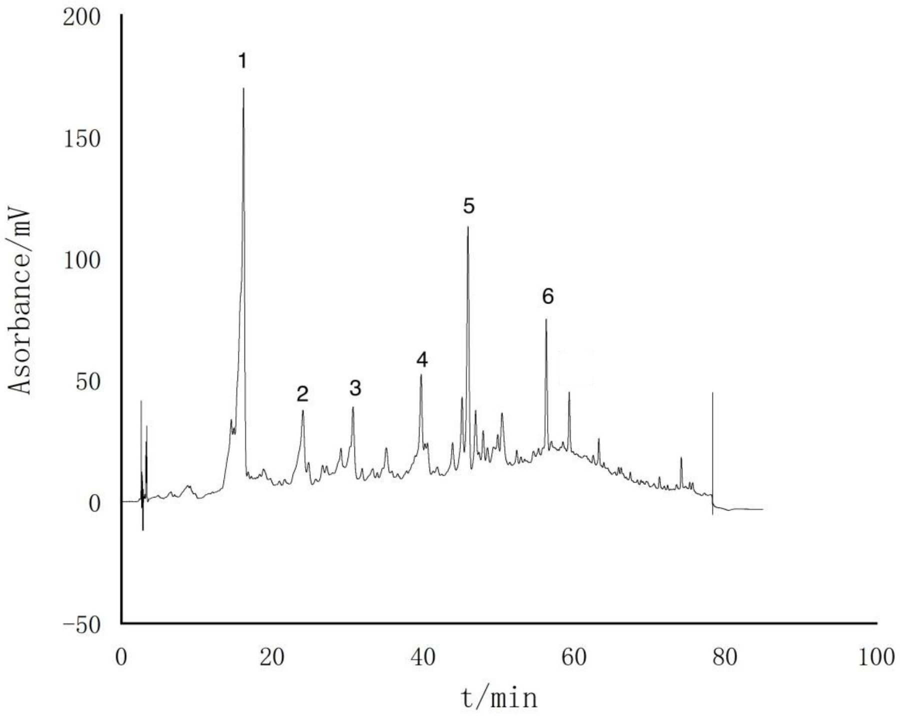

Figure S1. HPLC chromatogram of *Hedysari Radix*.

Table S1. Identified chemical components of *Hedysari Radix* extract by HPLC.

Peak No.	Compound Name	*Retention Time (min)*	Identification Method	Remark
1	Ferulic acid	16.8	Compared with authentic standard	Identified
2	Isoferulic acid	24.2	Compared with authentic standard	Identified
3	Vanillic acid	28.5	Compared with authentic standard	Identified
4	Ononin	36.8	Compared with authentic standard	Identified
5	Formononetin	47.0	Compared with authentic standard	Identified
6	Formononetin-7-O-β-D-glucoside	58.2	Compared with authentic standard	Identified

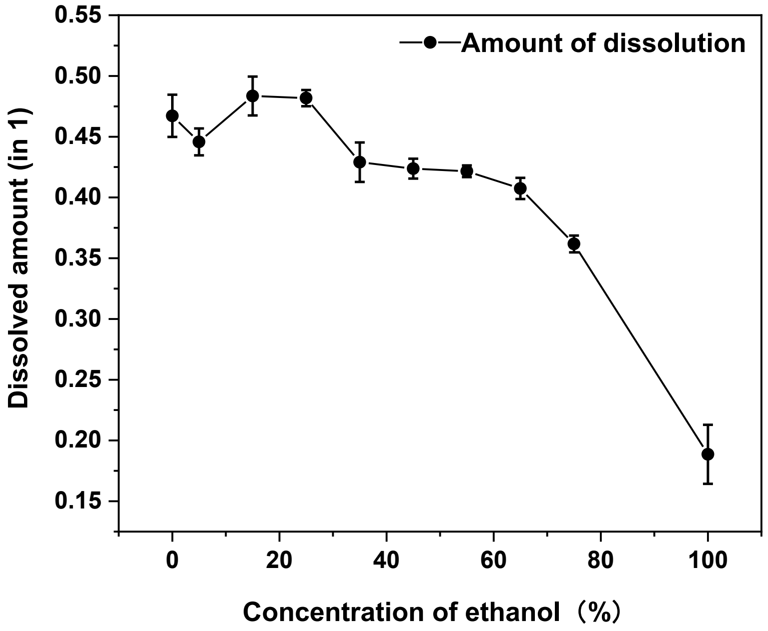

Figure S2. Dissolved amounts of Hedysari Radix in different ethanol percentages (0-100%).

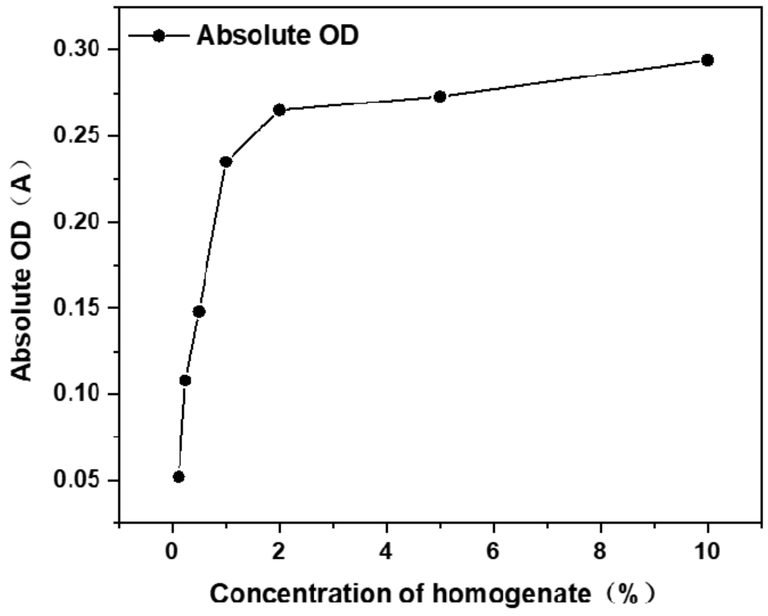

Figure S3. Absolute OD plots of different homogenate concentrations.
